# Evidence for Endemic Chikungunya Virus Infections in Bandung, Indonesia

**DOI:** 10.1371/journal.pntd.0002483

**Published:** 2013-10-24

**Authors:** Herman Kosasih, Quirijn de Mast, Susana Widjaja, Primal Sudjana, Ungke Antonjaya, Chairin Ma'roef, Silvita Fitri Riswari, Kevin R. Porter, Timothy H. Burgess, Bachti Alisjahbana, Andre van der Ven, Maya Williams

**Affiliations:** 1 United States Naval Medical Research Unit Two, Jakarta, Indonesia; 2 Health Research Unit, Faculty of Medicine, Universitas Padjadjaran, Bandung, Indonesia; 3 Department of General Internal Medicine, Radboud University Nijmegen Medical Centre, Nijmegen, the Netherlands; 4 Internal Medicine Department, Faculty of Medicine, Universitas Padjadjaran, Hasan Sadikin Hospital, Bandung, Indonesia; 5 Department of Parasitology, Faculty of Medicine, Universitas Padjadjaran, Bandung, Indonesia; Centers for Disease Control and Prevention, United States of America

## Abstract

Chikungunya virus (CHIKV) is known to cause sporadic or explosive outbreaks. However, little is known about the endemic transmission of CHIKV. To ascertain the endemic occurrence of CHIKV transmission, we tested blood samples from patients with a non-dengue febrile illness who participated in a prospective cohort study of factory workers in Bandung, Indonesia. From August 2000 to June 2004, and September 2006 to April 2008, 1901 febrile episodes occurred and 231 (12.2%) dengue cases were identified. The remaining febrile cases were evaluated for possible CHIKV infection by measuring anti-CHIKV IgM and IgG antibodies in acute and convalescent samples. Acute samples of serologically positive cases were subsequently tested for the presence of CHIKV RNA by RT-PCR and/or virus isolation. A total of 135 (7.1%) CHIKV infections were identified, providing an incidence rate of 10.1/1,000 person years. CHIKV infections were identified all year round and tended to increase during the rainy season (January to March). Severe illness was not found and severe arthralgia was not a prominently reported symptom. Serial post-illness samples from nine cases were tested to obtain a kinetic picture of IgM and IgG anti-CHIKV antibodies. Anti-CHIKV IgM antibodies were persistently detected in high titers for approximately one year. Three patients demonstrated evidence of possible sequential CHIKV infections. The high incidence rate and continuous chikungunya cases in this adult cohort suggests that CHIKV is endemically transmitted in Bandung. Further characterization of the circulating strains and surveillance in larger areas are needed to better understand CHIKV epidemiology in Indonesia.

## Author Summary

Chikungunya is one of the neglected diseases. It has only attracted attention during outbreaks, in particular, the large epidemics in the Indian Ocean in 2005–2006. To our knowledge, there has never been any surveillance to determine the transmission of this virus among humans in non-outbreak settings. Such surveillance is particularly important because it will provide a better estimate of the disease burden and valuable information on how this virus is maintained outside outbreaks. Our study, conducted between 2000 and 2008 in Bandung, West Java, Indonesia, yielded several important findings: 1. Chikungunya is an important cause of fever among adults in Bandung, Indonesia. 2. The clinical symptoms are mostly mild and short lasting. 3. In addition to previously described epidemiological features involving episodic outbreaks, it is also continuously transmitted throughout the year. 4. A few patients may have experienced more than one chikungunya virus infection. 5. Only the Asian genotype was found and not the East Central South African genotype that was responsible for the 2005 outbreak in the Indian Ocean. 6. The persistence of IgM for a long period after illness may complicate the interpretation of laboratory results.

## Introduction

Chikungunya virus (CHIKV) is an arthropod-borne virus belonging to the genus *Alphavirus* in the family *Togaviridae* [1]. CHIKV causes an acute illness similar to dengue, characterized by fever, headache, nausea, vomiting, abdominal pain, myalgia, rash and arthralgia [2]. Arthralgia of the large joints may be severe and long lasting [3]. CHIKV was first identified in Tanzania in 1952 [4]. Over the next decades, it remained a relatively rare disease causing mostly small outbreaks in both Africa and Asia [2]. This changed dramatically after a mutation in the CHIKV E1 glycoprotein gene *(A226V)* occurred. This mutation enhanced the infectivity of the virus and its transmission by *Aedes albopictus* [5]. In 2005, the mutated CHIKV spread from the Indian Ocean where it produced large epidemics in India, Southeast Asia and Italy [6]–[9].

CHIKV in Africa is predominantly maintained in an inter-epidemic sylvatic cycle in which the virus resides in wild primates and mosquitoes such as *Aedes furcifer-taylori* and *Aedes africanus*. In Asia, *Aedes aegypti*, an anthrophophilic mosquito that lives in proximity with humans, has been the most significant vector [10]. In contrast, for the mutated CHIKV that caused the recent epidemic in the Indian Ocean, *Aedes albopictus* is the main vector [5].

In Indonesia, chikungunya was first reported in 1982 in East Sumatera. It then spread to other islands including Java, Kalimantan, Bali, Flores and Sulawesi [11]. After a hiatus of 15 years, sporadic outbreaks were reported simultaneously in several provinces on the island of Java in 2000–2002 [11]. Since then, clusters of cases have been reported sporadically from several provinces although the total number of cases reported has never exceeded 5,000 per year [12], [13]. This number should be interpreted with caution, however, because similarities in symptoms between dengue and chikungunya [2] and logistic constraints in viral diagnostics in Indonesia [14] may have resulted in a gross underestimation of the incidence of chikungunya [15]. To better define the disease burden of chikungunya, active surveillance during non-outbreak periods is necessary. However, to our knowledge, no such studies have been conducted elsewhere. Therefore, to determine CHIKV transmission during inter-epidemic periods and the epidemiology of CHIKV infections in Indonesia, we analyzed the demographic, clinical and virological data collected from non-dengue acute febrile patients participating in a prospective adult cohort dengue study that was conducted in Bandung, West Java, Indonesia from 2000–2004 and 2006–2008.

## Materials and Methods

### Study design

This study was a part of “An epidemiology study of dengue and dengue hemorrhagic fever in adults”, approved by the Institutional Review Board of NAMRU#2, Jakarta (IRB#30855 and N2.2006.0001) and the National Institute of Health Research and Development (NIHRD), Ministry of Health, Indonesia (KS 02.02.2.1.2181, KS 02.01.2.1732 and KS.02.01.2.1.2776) in compliance with all U.S. Federal Regulations governing the protection of human subjects. Details of the study design are described elsewhere [16]. In brief, it was a textile factory-based prospective cohort study conducted in Bandung, West Java, Indonesia, a city that has more than 2 million inhabitants. The study was conducted in two phases, 2000–2004 and 2006–2008. Phase 1 was carried out in factories A and B, and phase 2 was carried out in factories A and C. A cohort of 2978 volunteers was maintained during the first phase and 2726 during the second phase with 44.5% of volunteers from cohort 1 also participating in cohort 2. All volunteers gave written informed consent prior to enrollment. During enrollment, demographic and health status data were obtained and baseline blood specimens were collected. On a quarterly basis, surveys were conducted, and blood samples were taken to examine the volunteers' dengue serological status. Between each survey, volunteers who experienced fever came to the factory clinic where a clinical evaluation was performed and acute and convalescent (at least 7 days apart) blood specimens were collected. Specimens were immediately tested for dengue using a battery of dengue diagnostic assays [16]. Patients were advised to be hospitalized at the discretion of the attending physician or if their platelet count was less than 150,000/mm^3^. Once a dengue infection was excluded, the samples were tested for evidence of CHIKV infection as described below. During phase 1 of the study, volunteers were not questioned about arthralgia and data concerning arthralgia was obtained when this symptom was a chief or other complaint during their illness or during post-illness serosurveys. During phase 2 of the study, volunteers were specifically asked about arthralgia. There were no other differences in symptom ascertainment between phase 1 and 2.

### Diagnosis of CHIKV infections

Convalescent sera from febrile volunteers who had been excluded as dengue cases were first tested to detect CHIKV IgM antibodies using enzyme-linked immunosorbent assay (ELISA). When positive, paired acute and convalescent sera were further tested for CHIKV IgM and IgG antibodies using an ELISA, and acute sera were processed for viral isolation and/or tested by RT-PCR to detect CHIKV genomes. CHIKV infection was confirmed when CHIKV genome or virus was detected, or when either seroconversion or a four-fold rise in titers of anti-CHIKV IgM and IgG antibodies was detected.

### CHIKV IgM and IgG ELISA

Serum samples were assayed for the presence of IgG and IgM antibodies against CHIKV using ELISA as previously described [14], [17]. The CHIK antigen was prepared from Vero E6 cell culture infected with CHIKV 23574, an Asian lineage virus isolated from CHIK infected patient. Uninfected Vero E6 cell culture was used as negative antigen. For detection of CHIK IgM, 96- well microtiter plates (Immulon 2, Dynex Technologies, Chantilly, VA) were coated with anti-human IgM antibodies (Kirkegaard and Perry, Gaithersburg, MD). Excess antibodies were washed with 0.1% Tween Phosphate-buffered saline (PBS). Serum was diluted 1∶100 in dilution buffer (PBS, 0.1% Tween-20, and 5% skim milk), and incubated at 37°C for one hour. Plates were then washed and antigens were added. After incubation, anti-CHIK hyperimmune mouse ascitic fluid and horseradish peroxidase-conjugated anti-mouse IgG (Kirkegaard and Perry, Gaithersburg, MD) were used to detect IgM specific to CHIKV. ABTS substrate was allowed to react for one hour and absorbance was determined at 415 nm. For the detection of CHIK specific IgG antibodies, a 96-well microtiter plate was coated directly with cell lysate antigens diluted in PBS. Horseradish peroxidase conjugated mouse anti-human IgG Fc (Kirkegaard and Perry, Gaithersburg, MD), and ABTS were used to detect bound antibody. The adjusted optical density value (OD) for each sample was determined by subtracting the OD obtained with the negative antigen from the OD obtained using the CHIK antigen. A sample was considered positive if its OD value exceeded the mean plus three standard deviations of the normal control sera. The endpoint of antibody titers was determined by testing ELISA-positive samples at serial two-fold dilutions starting from 1∶100. The highest dilution showing a positive result was considered the endpoint titer. It has previously been established that our CHIK immunoassay does not show immunoreactivity against Ross River virus [17].

The kinetics of IgM and IgG antibodies to CHIKV were analyzed using specimens from the first nine patients who had complete serial quarterly serosurvey sera for two years.

### Virus isolation and CHIKV RT-PCR

Acute sera from cases diagnosed by seroconversion or a four-fold increase of anti-CHIKV IgM and IgG antibody titers were processed for virus isolation and analysis by RT-PCR. The methods used to perform these assays have been described previously [14], [17]. For virus isolation, serum samples were diluted 1∶10 in PBS and applied to confluent monolayers of C6/36 cells in 24-well culture plates (Corning, New York). The plates were centrifuged at 400*g* for 45 minutes and then 1 ml of medium (MEM) added. The plates were then incubated at 30°C for 14 days and observed daily for evidence of cytopathic effects (CPE). At the end of 14 days or upon recognition of CPE, cells were removed from the plates and evaluated for the presence of virus by standard immunofluorescence assay using anti-CHIK hyperimmune mouse ascitic fluid and FITC conjugated anti-mouse IgG (Kirkegaard and Perry, Gaithersburg, MD). For RT-PCR, viral RNA was extracted using a QIAamp Viral RNA Isolation Kit (QIAGEN, Hilden, Germany). RNA was then used in a nested RT-PCR assay using JM1 (5′ GCAGAC GCAGAGAGGGCCAG 3′; bp 1,201 to 1,220) and JM2 (5′ CGTGCTGCAAGG TAGTTCTC 3′; bp 1,440 to 1,421) primers. A second nested PCR was performed using the product from the first reaction and primers JM3 (5′ GCTATTTGTAAGAAC GTCAG 3′; bp 1,221 to 1,240) and JM4 (5′ TACCGTGCTGCGGTCGGGAA 3′; bp 1,420–1,401). Amplified PCR products were resolved by electrophoresis on a 2% agarose gel and visualized using ethidium bromide.

### Genomic sequencing

Sequencing of the structural polyprotein coding region of 20 chikungunya virus isolates was performed (Genbank accession numbers: KC879559–KC879578), using primers that were previously described [18]. Cycle sequencing reactions were conducted using the BigDye 3.3 Terminator Ready Reaction mix (Applied Biosystems, Carlsbad, California). Cycle sequencing was performed at least twice per primer per sample. The sequencing products were separated from unbound dye using BigDye X-Terminator (Applied Biosystems) and analyzed on an ABI 3130 XL Genetic Analyzer (Applied Biosystems).

Sequence data analysis was performed using Sequencher 3.1 (Genecodes, Ann Arbor, MI) with the default parameters to improve the overall sequence quality. ClustalX 2.0.9 [19] was used to perform multiple sequence alignment. The alignment parameters used were 50 points penalty for gap opening, 2 point penalty for gap extension, and all gaps were reset before each alignment. Several reference sequences were used for the alignment: six Asian genotype viruses (among them a vaccine strain), five East/Central/South African (ECSA) genotype viruses, and one West African genotype virus. After obtaining the alignment, excess sequence (outside the coding region for the structural proteins) was discarded. The phylogenetic tree was constructed using the neighbor-joining method [20] with MEGA4 software [21]. The distance model used was the Kimura 2 parameter model to correct for multiple substitutions and to account for unequal transition/transversion ratio. The tree was constructed with 1000 bootstrap replicates.

### Statistical analysis

Descriptive data (mean age and standard deviation) were analyzed using STATA version 9.0 (StataCorp 2005, College Station, TX).

## Results

### Study population

A total of 4380 volunteers were enrolled into the study, consisting of 1324 volunteers who joined from the beginning of this study (August 2000), 1654 who discontinued their participations after the first phase (June 2004) and 1402 new enrollees in the second phase (September 2006 to April 2008). The mean (SD) age and age range of volunteers at enrollment were 37.1 (±7.7) and 18 to 66 years old. A higher proportion of the study population was male (ratio 1.85∶ 1).

### Chikungunya cases

#### The proportion and incidence rate of chikungunya cases

**Figure 1:**
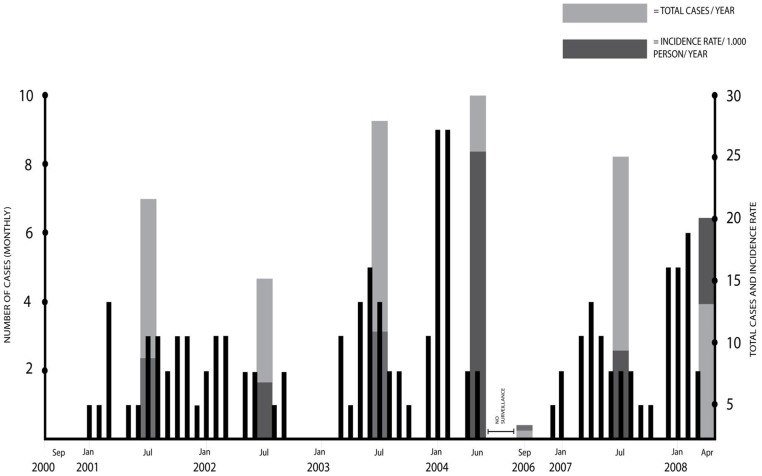
Monthly, annual total number and annual incidence rates for laboratory confirmed chikungunya (CHIK) cases. The number of CHIK cases per month is represented by skinny black rectangles. The total number of cases for a year is indicated by light gray rectangles and the incidence rate for a year is indicated by dark gray rectangles. The rectangles representing annual data are placed at the midpoint (July) for their respective year.

A total of 1431 acute febrile episodes (AFE) occurred among 2978 volunteers in the first phase of the study, which lasted for 47 months. CHIKV infection was identified in 96 (6.7%) of these AFE, yielding a yearly incidence rate of 10.1 per 1,000 persons. During the second phase of the study in which 2726 volunteers were followed for 20 months, 39 chikungunya cases were diagnosed among 470 AFE, resulting in a slightly higher percentage and incidence rate (8.3% and 10.3/1,000 persons/year). In total, the percentage and incidence rate were 7.1% and 10.1/1,000 (persons/year), respectively. The number of chikungunya cases per month over the years and the total number of cases per year are shown in figure 1. The number of cases remained relatively stable over the years, except for two peaks in early 2004 and 2008, when chikungunya accounted for 11.2% and 11.7% of the AFE. In contrast, no cases were found in the second half of 2000 and only one case in the last four months of 2006. Cases were found almost all year round, however November had the lowest number of cases and cases tended to increase during the wet season from January to March. Chikungunya cases resided throughout the city without apparent clustering by residence although the majority of cases were from the subdistricts near the factories where most workers lived (data not shown).

#### Diagnosis of CHIKV infections, antibody kinetics and virus sequencing

In 69 (51.1%) of the 135 CHIKV infections, the diagnosis of acute CHIKV infection was confirmed by positive RT-PCR, viral isolation and serology results. In 47 (34.8%) cases the diagnosis was confirmed by positive RT-PCR and serology results, while 19 (14.1%) cases only had serological evidence of CHIK infection. Of these 19 cases, 10 were negative for RT-PCR and isolation, eight did not have RT-PCR performed and one did not have viral isolation performed due to an insufficient volume of serum.

**Figure 2:**
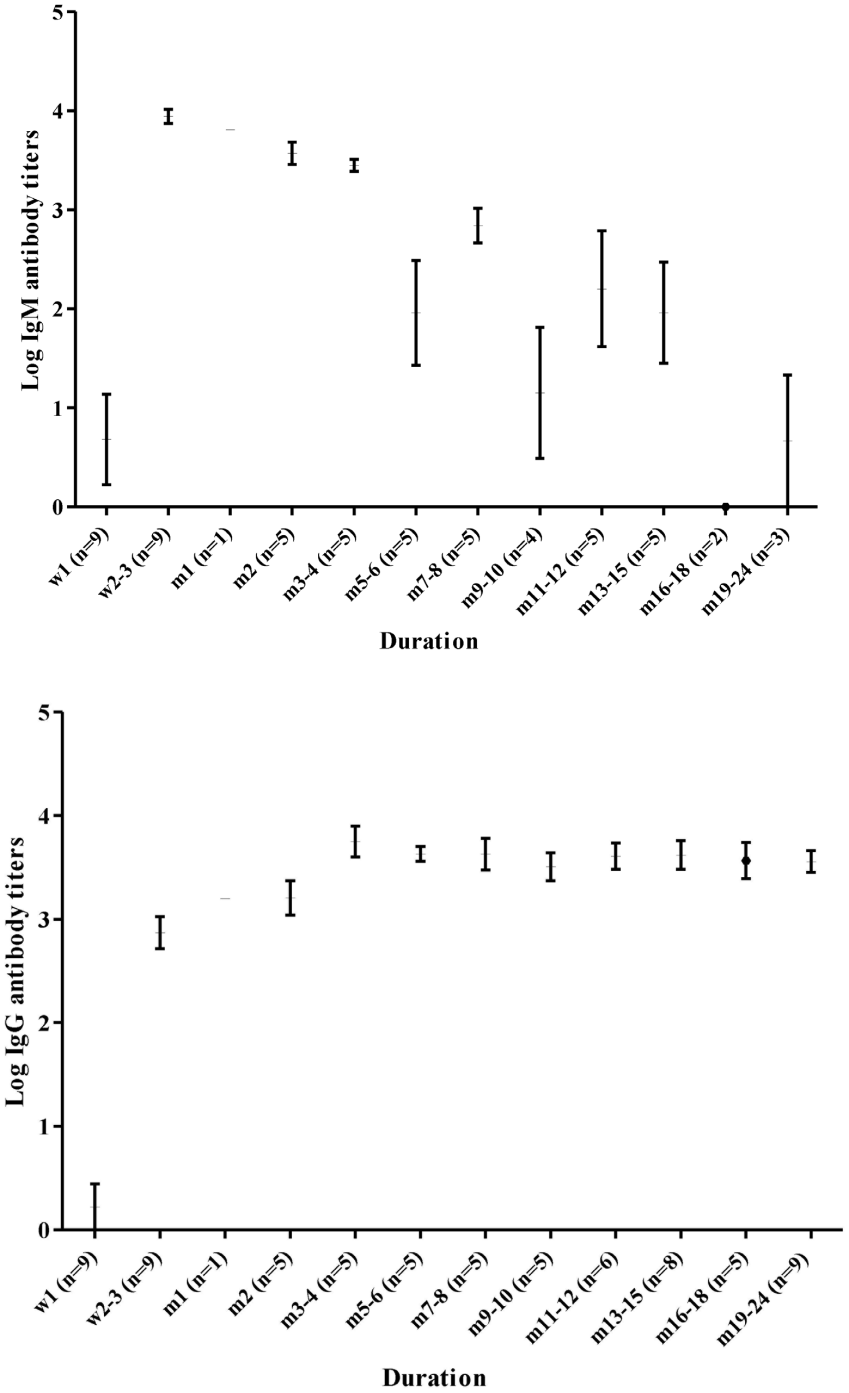
Immunokinetics of anti-chikungunya virus IgM (top) and IgG (bottom). The mean +/− SD titer for each timepoint (W = weeks, M = months) after illness onset. N = the number of samples for a given timepoint.

Post-illness sera from nine patients, who had serial specimens taken during serosurveys over two years, were tested to evaluate the kinetics of CHIKV IgM and IgG antibodies (figure 2). These nine patients did not have any anti-CHIKV IgM or IgG antibodies in their pre-illness samples and IgM antibodies were undetectable in the acute specimen of seven of these patients. The two patients in which IgM was detectable in the acute sample came to the clinic later in disease on day five and seven of illness and had anti-CHIKV IgM titers of 400 and 3200 respectively. In convalescent and late convalescent sera, titers increased strongly, followed by a subsequent slow decline thereafter. In most patients (8/9), IgM antibodies were detectable for a long period after acute infection, ranging from 5–22 months. In one patient, the IgM titer became undetectable three months after the onset of illness. The demographic and clinical findings of this patient did not differ from the rest. IgG antibodies were not detected in 8 of 9 acute samples while one sample had a titer of 100. In convalescent sera, which were collected 2–3 weeks after onset of fever, IgG antibodies were detected at low titers (200–800) in 7 of 9 patients and at high titers (1600 and 3200) in two patients. IgG titers peaked (6400 to 1∶25600 dilution) at 3–4 months after the onset of illness, then decreased slightly and remained stable at high titers (1600 to 3200) for two years after illness.

**Figure 3:**
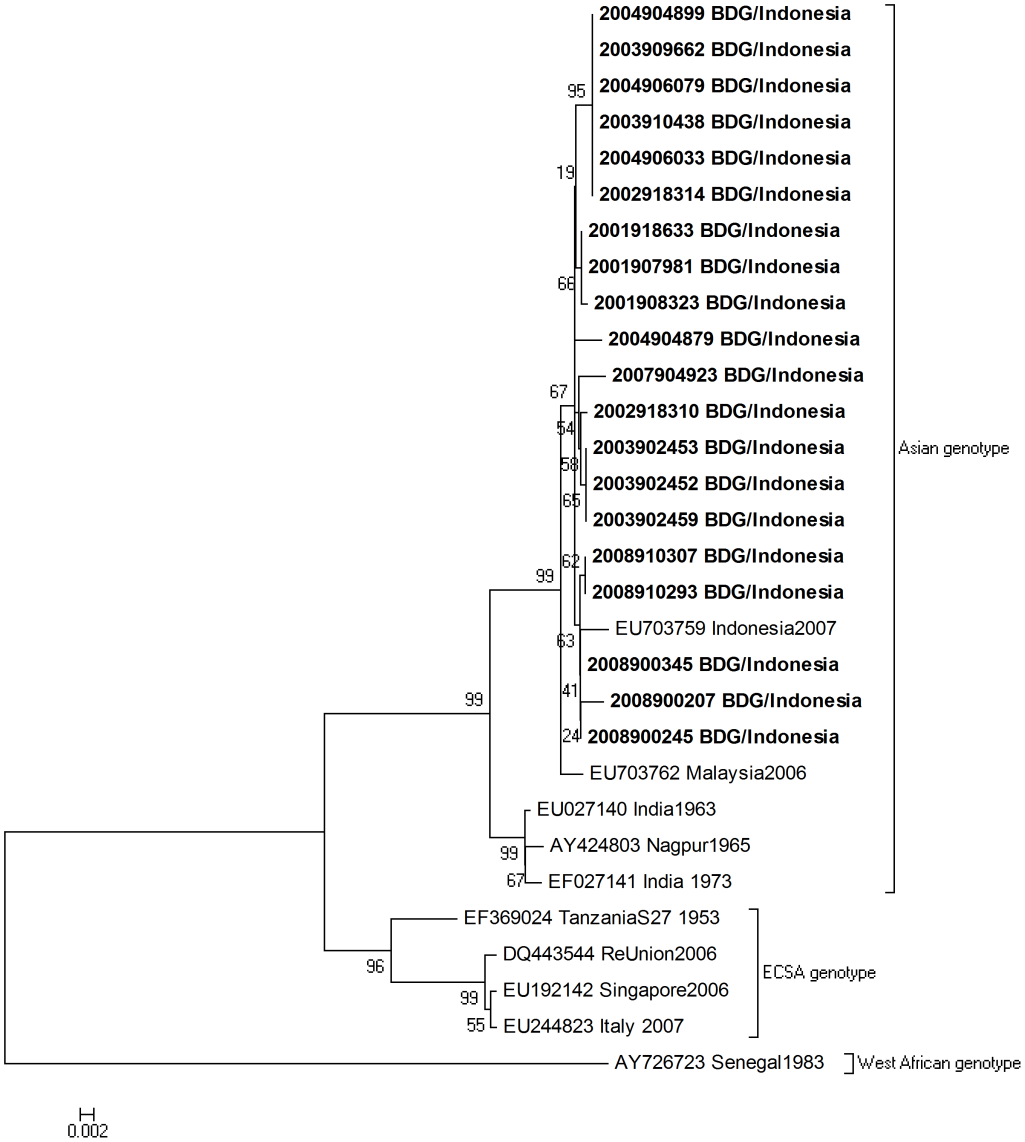
Phylogenetic analysis of the CHIKV isolates. Neighbor-joining tree of the structural polyprotein coding region of CHIKV. The sequences obtained in this study are highlighted in bold. Numbers indicate bootstrap values for the groups to the right.

CHIKV could be isolated from specimens collected until day 4 of the illness whereas RNA was detectable by RT-PCR until 6 days post illness onset. Sequencing analysis was performed on 20 isolates from the first and second phases of the study. All isolates had alanine at position 226 in the E1 gene. Nucleotide similarity between these isolates was >99.4%, and amino acid similarity was >99.7%. A phylogenetic tree was constructed based on 1320 bases of the structural polyprotein coding region. All samples sequenced from this study clustered together and belong to the Asian genotype (figure 3).

#### Clinical manifestations

Most patients came to the clinic on day two or three of fever (57 and 56 patients out of 135 patients, respectively). Table 1 lists their clinical manifestations. The most frequent were myalgia (92.5%), headache (88.8%), and arthralgia (38.9% during the first phase and 87.2%, during the second phase). Among 34 patients with arthralgia in the second phase of the study, the most commonly involved joints were the knees (85.3%), shoulders (64.7%), smaller joints and elbow (58.8%). The initial working diagnoses made by clinicians during enrollment at the health centers in cases with laboratory confirmed chikungunya included undifferentiated fever (71.1%), dengue fever (12.6%), upper respiratory tract infection (8.1%), typhoid fever (3%), chikungunya fever (1.5%), measles and gastroenteritis (each 0.7%). Clinical manifestations were mostly mild and no severe cases occurred. Seven patients (5.2%) were hospitalized because their platelet level was under 150,000/mm^3^, five from the first cohort and two from the second cohort. As chikungunya diagnostic assays were not available at the hospitals, discharged clinical diagnoses were acute viral infection and dengue fever in 5 and 2 patients, respectively. Approximately one third (32.6%) of patients did not skip work at all, 36.3% were absent for 1 to 3 days, 19.3% for 4 to 6 days and 11.9% for more than a week. The average number of days absent was 2.7 days.

**Table 1:** Signs, symptoms and laboratory results for CHIK cases.

Symptoms	N	% Pos
Myalgia	124/134	92.5
Headache	119/134	88.8
Arthralgia		
1^st^ phase	37/95	38.9
2^nd^ phase	34/39	87.2
Nausea	71/134	53.0
Retro-orbital pain	51/134	38.1
Cough	37/134	27.6
Abdominal Pain	33/134	24.6
Sore throat	31/134	23.1
Coryza	31/134	23.1
Rash	17/134	12.7
Vomiting	15/134	11.2
Diarrhea	14/134	10.4
Leukopenia(<4000/mm^3^)	20/134	14.9
Thrombocytopenia (<150,000/mm^3^)	15/134	11.2

### Evidence of possible recurrent CHIKV infections

We identified two patients with a laboratory confirmed acute CHIKV infection in whom serology results on a blood sample collected three months before illness suggested a previous CHIKV infection. In the first volunteer, an acute CHIKV infection was confirmed by RT-PCR, virus isolation and serology. In the second patient, CHIKV infection was confirmed by RT-PCR and serology (Table 2). Both patients reported fever, headache and myalgia, but no arthralgia. We also identified a 34-year-old male for whom we had evidence of two possible chikungunya episodes during our study. The first episode was in June 2002 when an acute CHIKV infection was confirmed by positive RT-PCR, virus isolation and IgM and IgG sero-conversion; the second episode occurred four and half years later and was confirmed by a four-fold rise in IgM (Table 2). For both episodes, dengue was excluded as all dengue diagnostic tests were negative. Clinical manifestations in both episodes were similar, including high fever, headache, sore throat, malaise and bilateral arthralgia. Results from hematology and chemistry tests did not show pathognomonic findings.

**Table 2:** Lab results for possible recurrent CHIKV infections.

ID Number	First infection		Second infection	
	Date of illness	Lab results	Date of illness	Lab results
005-1411	unknown	Pre-illness specimen	4 JAN2003	Positive RT-PCR and
		(21 SEP 2002)		Isolation
		CHIKV IgM: 400		CHIKV IgM: 800 to 400
		CHIK IgG: 800		CHIKV IgG: 6400 to 6400
005-2048	unknown	Pre-illness specimen	6 MAR2001	Positive RT-PCR
		(25 NOV2000)		CHIKV IgM: 100 to 400
		CHIKV IgM: 400		CHIKV IgG: 3200 to 6400
		CHIK IgG: 800		
005-1449	11 JUN 2002	Positive RT-PCR and	19 DEC2006	Negative RT-PCR and
		Isolation		Isolation
		CHIKV IgM: neg to 6400		CHIKV IgM: 400–1600
		CHIKV IgG: neg to 100		CHIKV IgG: 1600–6400

## Discussion

Our studies, conducted between 2000 and 2008 in a large town in West Java, Indonesia, revealed several important epidemiological findings, including: 1. Among adults, CHIKV was an important cause of acute febrile illness. 2. CHIKV infections did not occur in epidemics as commonly reported, but were found throughout the year. 3. The clinical symptoms of CHIKV infection in this cohort were mostly mild and short-lived. 4. CHIKV infections were caused by the Asian genotype and not by the mutated East Central South African strain (ECSA), although only a limited number of samples were genotyped. 5. The persistence of IgM for a long period after illness may complicate the interpretation of laboratory results, and finally 6. We found evidence of possible recurrent CHIKV infections. We have previously reported an incidence rate of acute dengue of 18.1 cases per 1,000 persons per year (15.9% of febrile episodes) in the same cohort in 2000 to 2002 [16] and our present findings show that the corresponding incidence rate of chikungunya in these years was 7.9 cases per 1,000 persons per year (6.9% of febrile episodes). In 2004 and 2008, the incidence rates for chikungunya were in the same range as those for dengue (data not shown).The overall prevalence of CHIK between August 2000 to June 2004 and September 2006 to April 2008 was 7.1% of febrile episodes, and the overall incidence rate during this time frame was 10.1 cases per 1,000 persons per year.

Another finding of our study was that chikungunya infections were generally mild and of short duration. Only two-thirds of cases requested medical leave from work, with most only requesting 2–3 days of leave. The percentage of chikungunya cases in our study that were hospitalized was significantly lower than for the dengue cases detected in our study (unpublished data). Additionally, during the first phase of our study, when data on arthralgia was not specifically asked, only 38.9% of volunteers reported it as a chief or other complaint, suggesting this symptom was minor or absent. Also, we did not find volunteers with prolonged illness or complications. Similar to our clinical findings, mild chikungunya cases were also reported among young migrant workers in Singapore during a 2008 outbreak [22]. This is in contrast to what was reported in previous outbreaks where debilitating arthralgia was a frequent symptom in acute disease, sometimes persisting for months to even years [23]–[25] and in the recent La Reunion and India outbreaks where severe disease with neurological involvement was also reported [26], [27].

Genotyping of the CHIKV in twenty patients showed that infections were caused by the Asian genotype and not the ECSA genotype, which was responsible for the 2005 outbreak in the Indian Ocean and has since then spread to India and Southeast Asia, causing unprecedented nationwide outbreaks in Malaysia, Singapore and Thailand [7]. The detection of the ECSA genotype has not been reported in Indonesia and the Asian genotype was the only genotype identified in Taiwan travelers returning from Indonesia in 2007–2008 [28] and in hospitalized patients in Surabaya in 2011 [15]. As ECSA genotype has been detected elsewhere in Southeast Asia [18], [29]–[31], this genotype may have circulated in Indonesia as well, but remains unidentified as routine chikungunya surveillance has not been established. Still little is known about differences in clinical presentation and epidemiology between infections caused by the Asian and the ECSA genotype. Our findings suggest that the illness caused by this Asian genotype is often relatively mild and that infections occur year round in Bandung, Indonesia. Interestingly, infections by the same Asian genotype that were reported in travelers returning from Indonesia [32] and during outbreaks in Indonesia [11], [17] appear to be associated with more severe disease. Selection bias, whereby chikungunya was only considered in those with more severe disease, and differences in the CHIKV strains that circulate during the inter-epidemic period and those causing outbreaks and/or severe illness might explain these differences. Another plausible explanation of mostly mild cases in our study was the young adult population in contrast to children or the elderly who commonly experience severe illness [33]–[35].

According to our literature review, CHIKV infections have commonly been associated with outbreaks [7] and many countries reported a long hiatus between outbreaks, for example 32 years in India, 15 years in Indonesia and 7 years in Malaysia [11], [36], [37]. To the best of our knowledge, our study is the first to report year round CHIKV infections over several years. As such, it provides a better understanding of how CHIKV is maintained in the population. It was speculated that unreported infections were also the cause of an outbreak in Malaysia in 2006 [36]. One of the reasons chikungunya is not reported is the difficulty in distinguishing chikungunya clinically from other infections, such as dengue [2], [38]. This may be especially challenging when arthralgia, which is considered a pathognomonic symptom of chikungunya, is not a prominent clinical feature. Moreover, chikungunya is generally perceived to occur only in outbreaks [39]. The limited sensitivity of currently available rapid diagnostic tests for chikungunya and the long persistence of CHIKV IgM antibodies may further complicate the correct diagnosis of chikungunya [14], [40]. Advanced diagnostic tools such as virus isolation and RT-PCR are generally only available in large hospitals or research institutions. In our study, chikungunya cases were identified almost every month throughout the year from 2001–2004 and 2006–2008. The peak of chikungunya cases was during or after the monsoon season when the population of *Aedes aegypti* is abundant. This finding is consistent with previous reports from other countries [41]–[43]. In addition, acute chikungunya infections were still detected, albeit at a lower frequency, during the dry season, suggesting that virus transmission was maintained during this period. This is not surprising as *Aedes aegypti* and *albopictus* were found abundantly in Bandung and elsewhere in Indonesia throughout the year despite extensive eradication efforts [11], [44]–[47]. Based on the data from this study, we did not observe any clustering or focused geographical transmission (data not shown).

One of the powerful features of our prospective cohort study was that volunteers were followed for several years. Therefore, we were able to observe the kinetics of CHIKV IgM and IgG antibodies longitudinally after infection. Our finding that IgM antibodies could be detected beyond one year was consistent with previous reports [41], [48]. In a returning traveler from La Reunion Island, IgM antibodies remained detectable after two years and this was associated with persistent arthralgia [48]. In our study, among nine patients observed, IgM antibodies disappeared between five months and 19 months (median of 10 months) after infection. Post-illness sera were collected during programmed serosurveys and not related to any febrile episodes, arguing against the possibility that the high titers of anti-CHIKV antibodies were induced by a nonspecific polyclonal activation after unrelated infections.

Persistence of IgM antibodies also has consequences for diagnostics. In the absence of virus culture or RT-PCR, IgM and IgG serology assays should be conducted using paired sera collected at least 10 days apart to confirm the increasing titers. We also identified three patients (2%) with symptomatic CHIKV infections with laboratory features suggestive of possible secondary chikungunya infection, which, to our knowledge, has not yet been reported. Indeed, the current dogma holds that CHIKV infection will provide life-long immunity [49]. In one of these three patients, the RT-PCR for CHIKV was negative and the diagnosis of an acute chikungunya infection was based on a rapid increase in both IgM and IgG. This rapid increase in IgM and IgG, which also may have resulted in rapid virus clearance, was different from the kinetics of IgM and IgG antibodies during primary infections. IgM antibodies in primary infections were often not detected in acute sera or only identified in low titers, followed by a rapid increase during convalescence, when IgG antibodies first became detectable. We could not perform sequence analysis to identify the differences between CHIKV isolates in primary and secondary infections because, in two cases, the first infections occurred prior to their participation in this study while in the third case, CHIKV could be isolated only from the first infection. Cross-reactivity with viruses belonging to the Semliki group is a potential explanation. However, Ross River and O'nyong nyong viruses have never been reported in Indonesia and our CHIKV IgM immunoassay does not show immunoreactivity with the Ross River virus [17]. In the absence of virological data from human cases or mosquitoes due to the scarcity of chikungunya research in Indonesia, we speculate that these three patients in Bandung were re-infected by different CHIKV strains. Further studies are needed to confirm this hypothesis, including close longitudinal observation of those who experienced first CHIKV infections and performance of plaque reduction neutralization tests, which were not available in our laboratory due to the BSL-3 containment requirement.

Our study has some limitations. First, data on arthralgia as a symptom during the first phase of the study was collected based on passive reports from patients during their visits. On the other hand, it may also provide us some information regarding the percentage of patients who consider arthralgia as a prominent symptom. Listing arthralgia as one of the subjective symptoms that should be routinely included in questionnaires during acute illness may unintentionally increase the likelihood of subjects endorsing this symptom. Second, as mentioned above, the diagnosis of previous infections in two cases and recurrent infection in one case was based solely on results obtained by ELISA. Detecting the presence of virus either by tissue culture isolation or RT-PCR, or performing plaque reduction neutralization assays on the serum samples would have provided more definitive evidence for recurrent infections

In conclusion, our findings provide an estimate of the disease burden of CHIKV infections in Bandung, Indonesia and especially provide new information on its endemic transmission during inter-epidemic periods. These data highlight the importance of considering chikungunya in the differential diagnoses of acute febrile illnesses. Further studies are required to determine the significance of persistent IgM antibodies and the relation to arthralgia, the possibility of repeat CHIKV infections and the differences between strains in their potential to cause severe illness and epidemic versus endemic transmission. In addition, national surveillance needs to be established to monitor for the possible introduction of the ECSA genotype into Indonesia, as the transmission of this genotype may have a greater impact on public health. Finally, our findings highlight the need for development of affordable and sensitive rapid antigen diagnostic tests for early diagnosis of CHIKV infections and the need for a vaccine, especially since vector control has been unsuccessful so far.

## Supporting Information

Checklist S1 **STROBE checklist.**
(DOCX)
