# Supplementary material for: Evidence for Endemic Chikungunya Virus Infections in Bandung, Indonesia
Source: PLoS Negl Trop Dis. 2013 Oct 24;7(10):e2483. doi: 10.1371/journal.pntd.0002483 (PMC3812099; doi:10.1371/journal.pntd.0002483)
Supplement: Checklist S1 — STROBE checklist. (DOCX) [file pntd.0002483.s001.docx]

**CHECKLIST 1: STROBE checklist**

|  | Item No | Recommendation |
| --- | --- | --- |
| **Title and abstract** | 1 | (*a*) Indicate the study’s design with a commonly used term in the title or the abstract: page 1 line 17-18 |
|  |  | (*b*) Provide in the abstract an informative and balanced summary of what was done and what was found: page 1 line 20-29 |
| Introduction | | |
| Background/rationale | 2 | Explain the scientific background and rationale for the investigation being reported: page 2 line 21 to page 3 line 13 |
| Objectives | 3 | State specific objectives, including any pre specified hypotheses: page 3 line 12 to 16 |
| Methods | | |
| Study design | 4 | Present key elements of study design early in the paper: page 3 line 27 |
| Setting | 5 | Describe the setting, locations, and relevant dates, including periods of recruitment, exposure, follow-up, and data collection: page 3 line 27 to page 4 line 1 |
| Participants | 6 | (*a*) Give the eligibility criteria, and the sources and methods of selection of participants. Describe methods of follow-up: page 4 line 2-11 |
|  |  | (*b*) For matched studies, give matching criteria and number of exposed and unexposed: N/A |
| Variables | 7 | Clearly define all outcomes, exposures, predictors, potential confounders, and effect modifiers. Give diagnostic criteria, if applicable: for diagnosing chikungunya infection the criteria is listed on page 4 line 13-19 |
| Data sources/ measurement | 8* | For each variable of interest, give sources of data and details of methods of assessment (measurement). Describe comparability of assessment methods if there is more than one group: N/A |
| Bias | 9 | Describe any efforts to address potential sources of bias: N/A |
| Study size | 10 | Explain how the study size was arrived at: page 3 line 30 to page 4 line 5, page 5 line 8-9 |
| Quantitative variables | 11 | Explain how quantitative variables were handled in the analyses. If applicable, describe which groupings were chosen and why: N/A |
| Statistical methods | 12 | (*a*) Describe all statistical methods, including those used to control for confounding: page 6 line 16-18 |
|  |  | (*b*) Describe any methods used to examine subgroups and interactions: N/A |
|  |  | (*c*) Explain how missing data were addressed: exclude from the calculation: page 16 table 1 |
|  |  | (*d*) If applicable, explain how loss to follow-up was addressed: N/A |
|  |  | (*e*) Describe any sensitivity analyses: N/A |
| Results | | |
| Participants | 13* | (a) Report numbers of individuals at each stage of study—eg numbers potentially eligible, examined for eligibility, confirmed eligible, included in the study, completing follow-up, and analysed: page 6 line 24-26, page 6 line 32 to page 7 line 1, page 7 line 19 |
|  |  | (b) Give reasons for non-participation at each stage: N/A |
|  |  | (c) Consider use of a flow diagram: N/A |
| Descriptive data | 14* | (a) Give characteristics of study participants (eg demographic, clinical, social) and information on exposures and potential confounders: page 6 line 26-28, page 6 line 33 to page 7 line 1 |
|  |  | (b) Indicate number of participants with missing data for each variable of interest: page 4 line 7-10, page 7 line 17-18, page 19 table 1 |
|  |  | (c) Summarise follow-up time (eg, average and total amount): page 6 line 33 and page 7 line 1 |
| Outcome data | 15* | Report numbers of outcome events or summary measures over time: page 6 line 31 to page 7 line 11 |
| Main results | 16 | (*a*) Give unadjusted estimates and, if applicable, confounder-adjusted estimates and their precision (eg, 95% confidence interval). Make clear which confounders were adjusted for and why they were included: N/A |
|  |  | (*b*) Report category boundaries when continuous variables were categorized: N/A |
|  |  | (*c*) If relevant, consider translating estimates of relative risk into absolute risk for a meaningful time period: N/A |
| Other analyses | 17 | Report other analyses done—eg analyses of subgroups and interactions, and sensitivity analyses: page 7 line 13 to page 9 line 2 |
| Discussion | | |
| Key results | 18 | Summarise key results with reference to study objectives: page 9 line 5-27, page 10 line 1-2, 10-11, 27-32, page 11, 7-9 |
| Limitations | 19 | Discuss limitations of the study, taking into account sources of potential bias or imprecision. Discuss both direction and magnitude of any potential bias: page 12, line 4-12 |
| Interpretation | 20 | Give a cautious overall interpretation of results considering objectives, limitations, multiplicity of analyses, results from similar studies, and other relevant evidence: page 9 line 24-28, page 10 line 6-8, page 11 line 28-33 |
| Generalisability | 21 | Discuss the generalisability (external validity) of the study results: N/A |
| Other information | | |
| Funding | 22 | Give the source of funding and the role of the funders for the present study and, if applicable, for the original study on which the present article is based: page 13 line 2-4 |

*Give information separately for exposed and unexposed groups.
